# Constrained future brightening of solar radiation and its implication for China's solar power

**DOI:** 10.1093/nsr/nwac242

**Published:** 2022-10-29

**Authors:** Yanyi He, Kun Yang, Martin Wild, Kaicun Wang, Dan Tong, Changkun Shao, Tianjun Zhou

**Affiliations:** Department of Earth System Science, Ministry of Education Key Laboratory for Earth System Modeling, Institute for Global Change Studies, Tsinghua University, Beijing 100084, China; Department of Earth System Science, Ministry of Education Key Laboratory for Earth System Modeling, Institute for Global Change Studies, Tsinghua University, Beijing 100084, China; National Tibetan Plateau Data Center, State Key Laboratory of Tibetan Plateau Earth System and Resource Environment, Institute of Tibetan Plateau Research, Chinese Academy of Sciences, Beijing 100101, China; Institute for Atmospheric and Climate Science, ETH Zürich, Zürich 8001, Switzerland; Sino-French Institute for Earth System Science, College of Urban and Environmental Sciences, Peking University, Beijing 100081, China; Department of Earth System Science, Ministry of Education Key Laboratory for Earth System Modeling, Institute for Global Change Studies, Tsinghua University, Beijing 100084, China; Department of Earth System Science, Ministry of Education Key Laboratory for Earth System Modeling, Institute for Global Change Studies, Tsinghua University, Beijing 100084, China; State Key Laboratory of Numerical Modeling for Atmospheric Sciences and Geophysical Fluid Dynamics, Institute of Atmospheric Physics, Chinese Academy of Sciences, Beijing 100029, China

**Keywords:** surface downward solar radiation, CMIP6 model, emergent constraint, future change, solar energy

## Abstract

As Earth's primary energy source, surface downward solar radiation (*R*_s_) determines the solar power potential and usage for climate change mitigation. Future projections of *R*_s_ based on climate models have large uncertainties that interfere with the efficient deployment of solar energy to achieve China's carbon-neutrality goal. Here we assess 24 models in the latest Coupled Model Intercomparison Project Phase 6 with historical observations in China and find systematic biases in simulating historical *R*_s_ values likely due to model biases in cloud cover and clear-sky radiation, resulting in largely uncertain projections for future changes in *R*_s_. Based on emergent constraints, we obtain credible *R*_s_ with narrowed uncertainties by ∼56% in the mid-twenty-first century and show that the mean *R*_s_ change during 2050–2069 relative to 1995–2014 is 30% more brightening than the raw projections. Particularly in North China and Southeast China with higher power demand, the constrained projections present more significant brightening, highlighting the importance of considering the spatial changes in future *R_s_* when locating new solar energy infrastructures.

## Teaser

We narrowed the uncertainties in the projection of surface downward solar radiation by ∼56% based on emergent constraints. We found that the constrained predictions present significant brightening in North and Southeast China with high power demand.

## INTRODUCTION

To keep global warming to <2°C by the end of this century, renewable energy resources, such as solar energy generated by solar photovoltaic (PV) systems, have become increasingly important in the total primary energy supply [1–3]. Surface downward solar radiation (*R*_s_, with a wavelength of 0.2–4.0 μm) is the most critical indicator to know about the PV potential. To meet the carbon-neutrality goal, China has invested heavily in PV systems in the last decade. By the end of 2021, the installed capacity of the PV systems nationwide reached 306 GW, ranking first in the world for six consecutive years since 2015. Moreover, China plans 3550 GW of installed PV capacity by 2060, accounting for 45% of the total power generation in China [4]. Thus, robust estimates of future trends and uncertainties in solar radiation are crucial for realizing this promise of energy structure optimization.

Historical ground observations reveal that *R*_s_ declined from the 1950s to the 1980s and increased thereafter, known as global dimming and brightening [5]. In China, observational data show significant decadal variations in *R*_s_ in China with a dimming during the 1960s to 1990s and flattening thereafter (Supplementary Fig. S1a) [5–9]. Prior studies showed that the models that participated in the Coupled Model Intercomparison Project Phase 6 (CMIP6) overestimate the global mean *R*_s_ and underestimate *R*_s_ trend in China [10,11], but the underlying drivers of the *R*_s_ bias in CMIP6 models remain to be explored further. The CMIP6 experiments offer a unique opportunity for *R*_s_ assessment and projections under various emission scenarios that reflect policy impacts and socio-economic risks [12,13]. However, uncertainties in climate projections can arise from internal climate variability, model uncertainty and scenario uncertainty [14–17]. The parametric and structural model uncertainty associated with the model's response to specified forcing agents explains a major part of uncertainty in mid- to long-term climate projection [18]. To improve the reliability of climate projection, emergent constraints (EC) provide a novel approach with a solid physical basis for narrowing the uncertainties of future climate projections through the combination of an ensemble of climate simulations with contemporary measurements [19,20]. While EC have served to narrow down the uncertainty in equilibrium climate sensitivity, atmospheric circulation pattern and air temperature projection, among others [19–26], how to constrain the *R*_s_ projection remains to be explored through looking for a robust constraint relationship of *R*_s_.

In this paper, we assess the performance of *R*_s_ simulations from 24 CMIP6 climate models and explore whether their *R*_s_ biases over China are triggered by model biases in simulating total cloud cover and aerosol radiative effects. Then, we use historical biases of models to constrain the future projections of *R*_s_ under three possible future scenarios with different shared socio-economic pathways (SSPs) (SSP1-2.6, SSP2-4.5 and SSP5-8.5) based on EC. Details of our analytical approach are in the ‘Methods’ section. Our results can provide the best estimate and confidence interval of the *R*_s_ projection over China in the mid-twenty-first century and have important implications for the investment and construction of PV systems in China.

## RESULTS

### Model bias in simulating historical *R*_s_

**Figure 1.**
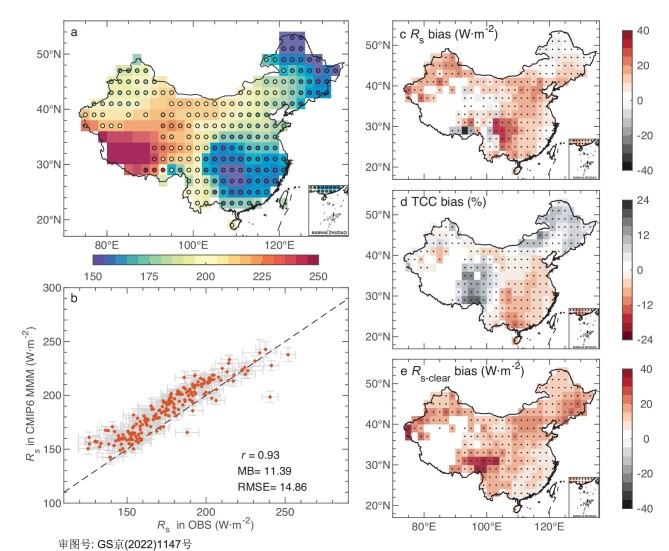
Maps of the climatology and model bias in climate simulations. (a) Spatial patterns of the climatology in surface downward solar radiation (*R*_s_, in W·m^−2^) from the ground-based observations (OBS, circle) and the multi-model mean (MMM, shading) of the CMIP6 historical all-forcing simulations averaged from 1961 to 2014 over 2° × 2° grids. (b) Grid-versus-grid comparison of the CMIP6 MMM with *R*_s_ observations in China. Orange dots and gray error bars show multi-year means and standard deviations of *R*_s_ for each grid, respectively. Correlation coefficient (*r*), mean bias (MB) and root-mean-square error (RMSE) are shown in the right-bottom. (c)–(e) Spatial patterns of multi-year mean biases in (c) *R*_s_, (d) total cloud cover fraction (TCC, in %) and (e) clear-sky surface downward solar radiation (*R*_s-clear_, in W·m^−2^) of the CMIP6 MMM against the ground-based observations averaged from 1961 to 2014. Black dots indicate that at least two-thirds of models agree on the sign of the mean bias in the CMIP6 MMM over those grids.

The multi-model mean (MMM) *R*_s_ of the CMIP6 historical all-forcing simulations presents a similar spatial pattern with the observations and the spatial correlation coefficient between the simulations and the observations over 2° × 2° grids is 0.93 (*P* < 0.05; Fig. 1a and b). Peaks of *R*_s_ mainly concentrate over the Tibetan Plateau with ∼250 W·m^−2^ (Fig. 1a), due to low air mass, short cloud liquid water pathways and low anthropogenic aerosol concentrations. Lower values of *R*_s_ mainly distribute in the east of China with the lowest value of ∼150 W·m^−2^ (Fig. 1a). The substantial reflection of clouds and the scattering and absorption of anthropogenic aerosols are responsible for low *R*_s_ over these regions [27,28].

However, the CMIP6 MMM *R*_s_ in the historical simulations is apparently overestimated at most grids (Fig. 1b), particularly in Northwest China and Southwest China (Fig. 1c). Compared with the observations, the CMIP6 MMM overestimates *R*_s_ by 11.39 ± 7.20 W·m^−2^ (mean ± 1 standard deviation) averaged in China from 1961 to 2014 (Fig. 1c and Table 1). The performance among CMIP6 models differs greatly, with the inter-model range of the *R*_s_ biases averaged in China over 30 W·m^−2^ (Table 1), which is larger than those averaged over the globe [11]. Most models exhibit a consistent pattern of overestimation with a spatial correlation of ∼0.7 against the CMIP6 MMM, and only 2 out of 24 models present negative biases in *R*_s_ (Table 1).

**Table 1.** Overview of the CMIP6 models used in this study, their horizontal grids, multi-year mean biases (MB) of surface downward solar radiation (*R*_s_, in W·m^−2^), total cloud cover fraction (TCC, in %) and clear-sky surface downward solar radiation (*R*_s-clear_, in W·m^−2^) referenced to station observations averaged over China from 1961 to 2014, spatial correlation coefficient (*r*) of the biases between individual model simulation and the multi-model mean (MMM) of the CMIP6 historical all-forcing simulations over the grids with observations. The rightmost column is the model weight (*w_i_*, in %) estimated for the weighted emergent constraint method (weight-EC).

		MB (*r*)			
CMIP6 models	Model grids	*R*_s_	TCC	*R*_s-clear_	*w_i_*
ACCESS-CM2	192 × 144	7.27 (0.81)	12.53 (0.93)	17.33 (0.81)	0.00
ACCESS-ESM1-5	192 × 145	4.09 (0.47)	11.39 (0.71)	20.24 (0.89)	19.93
AWI-CM-1–1-MR	384 × 192	14.31 (0.72)	–4.80 (0.88)	14.58 (0.86)	0.00
BCC-CSM2-MR	320 × 160	8.27 (0.72)	–7.32 (0.87)	17.68 (0.86)	0.00
CAS-ESM2-0	256 × 128	–1.18 (0.51)	–1.21 (0.64)	14.32 (0.86)	35.56
CESM2-WACCM	288 × 192	13.78 (0.70)	2.95 (0.92)	16.93 (0.70)	0.05
CMCC-ESM2	288 × 192	10.19 (0.72)	–0.06 (0.91)	22.24 (0.89)	0.07
CanESM5	128 × 64	13.45 (0.40)	–10.27 (0.70)	3.72 (0.48)	0.04
EC-Earth3-Veg-LR	320 × 160	10.49 (0.65)	2.62 (0.96)	12.42(0.84)	0.01
EC-Earth3-Veg	512 × 256	13.10 (0.58)	1.50 (0.93)	12.00 (0.88)	0.01
EC-Earth3	512 × 256	13.43 (0.58)	2.15 (0.93)	12.83 (0.76)	0.02
FGOALS-g3	180 × 80	27.72 (0.78)	–7.84 (0.76)	21.80 (0.87)	0.00
GFDL-ESM4	288 × 180	5.11 (0.83)	5.43 (0.95)	14.47 (0.88)	14.21
IITM-ESM	192 × 94	4.48 (0.53)	8.96 (0.82)	21.55 (0.88)	1.47
IPSL-CM6A-LR	144 × 143	22.50 (0.54)	–11.21 (0.89)	14.64 (0.43)	0.00
KIOST-ESM	192 × 96	–2.80 (0.74)	9.26 (0.91)	21.08 (0.88)	27.36
MIROC6	256 × 128	13.17 (0.88)	–10.50 (0.83)	28.03 (0.83)	0.00
MPI-ESM1-2-HR	384 × 192	14.81 (0.72)	–4.33 (0.88)	14.99 (0.87)	0.00
MPI-ESM1-2-LR	192 × 96	7.29 (0.71)	–0.18 (0.84)	14.83 (0.85)	0.83
MRI-ESM2-0	320 × 160	22.61 (0.53)	–7.33 (0.88)	13.05 (0.85)	0.00
NESM3	192 × 96	10.32 (0.64)	–1.44 (0.67)	3.07 (0.69)	0.11
NorESM2-LM	144 × 96	14.41 (0.77)	–6.46 (0.80)	18.56 (0.80)	0.00
NorESM2-MM	288 × 192	18.70 (0.74)	–2.73 (0.86)	19.14 (0.73)	0.00
TaiESM1	288 × 192	7.90 (0.78)	4.48 (0.96)	19.66 (0.92)	0.33
MMM	90 × 180	11.39	–0.60	16.21	–

### Drivers of model bias in historical *R*_s_

Clouds and aerosols have been widely regarded as the main controlling factors for changes in *R*_s_ [29,30]. The former can regulate *R*_s_ by reflecting solar radiation [31] and the latter also play a critical role in *R*_s_ changes due to their scattering and absorption of insolation, especially in regions with severe air pollution [32]. Although models have made significant progress in key physical processes of climate change, the biases in simulating these key processes may still be the major contributors to the biases of *R*_s_. The overall pattern of the total cloud cover fraction (TCC) can be simulated with a spatial correlation of 0.81 (*P* < 0.05) against the observed TCC in China (Supplementary Fig. S2a and b) but with evident spatial heterogeneities. For instance, models underestimate TCC in North China and Southeast China but overestimate in Northeast China, Northwest China and the Tibetan Plateau (Fig. 1d). The spatial pattern of the TCC bias is rather robust among the 24 models and the average value of the spatial correlation with the CMIP6 MMM is ∼0.85 (Table 1). The spatial pattern of the TCC bias in the CMIP6 MMM matches well with that of the *R*_s_ bias (Fig. 1c and d), with a spatial correlation of –0.51 (*P* < 0.05).

The historical simulations of clear-sky surface downward solar radiation (*R*_s-clear_) are also evaluated to identify the impact of aerosols on the simulated *R*_s_. First, the CMIP6 MMM *R*_s-clear_ exhibits a similar pattern as observations with a spatial correlation of 0.94 (*P* < 0.05; Supplementary Fig. S2c and d) but it is positively biased in China, especially in Northeast China and the Tibetan Plateau (Fig. 1e). This pattern of the *R*_s-clear_ bias is robust and consistent among the 24 models with a spatial correlation of ∼0.80 on average against the CMIP6 MMM (Table 1). Second, the simulated *R*_s-clear_ shows an excessive decline from 1961 to 2014, while both ground-based observations and satellite measurements show an increase after 2008 (Supplementary Fig. S1c). This is probably because the CMIP6 models significantly overload the decadal changes in anthropogenic aerosols in China [33].

**Figure 2.**
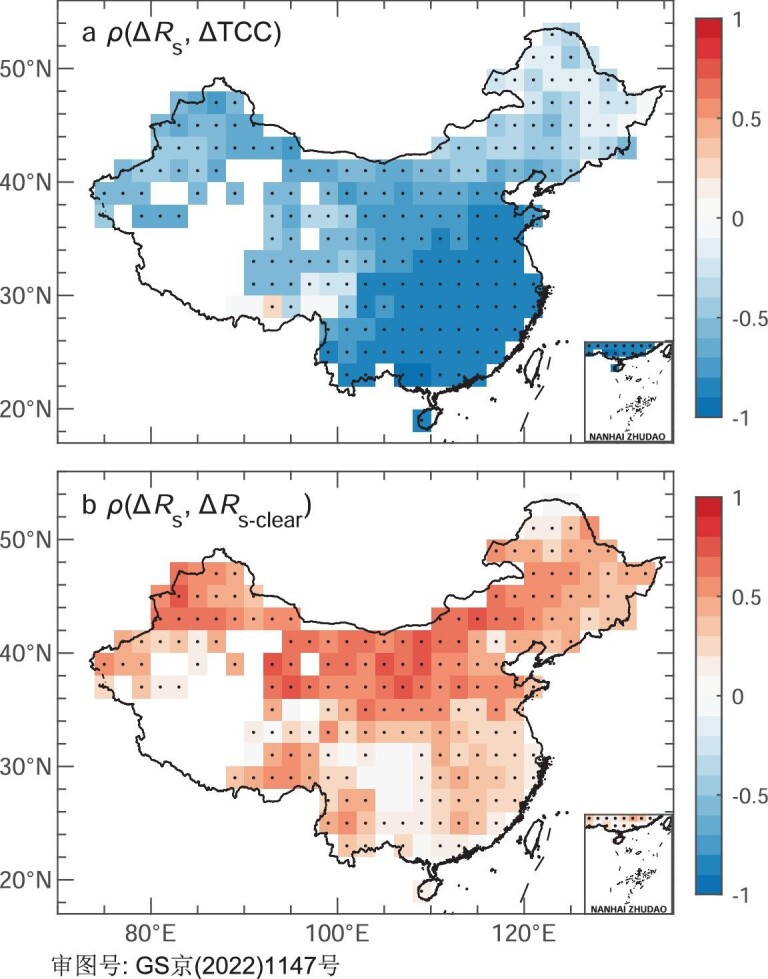
Sensitivities of model bias in surface downward solar radiation. (a) and (b) Spatial patterns of the partial coefficients (*ρ*) of the annual multi-model mean biases of the CMIP6 historical all-forcing simulations in surface downward solar radiation (Δ*R*_s_) against those in (a) total cloud cover fraction (ΔTCC) and (b) clear-sky surface downward solar radiation (Δ*R*_s-clear_) during 1961–2014. Black dots indicate a significance level of 0.05.

Figure 2 shows the sensitivities of model bias in *R*_s_ to TCC and *R*_s-clear_ biases, where the *R*_s_ bias has a larger partial correlation coefficient for the TCC bias in the south and the *R*_s-clear_ bias in the north. Overall, the bias of *R*_s_ in the CMIP6 MMM at the spatio-temporal scale can be explained by the combined effects of the simulated biases in TCC and *R*_s-clear_. In North China and Southeast China, the underestimation of TCC with high sensitivity to *R*_s_ and the overestimation of *R*_s-clear_ with low sensitivity to *R*_s_ jointly result in the positive bias of *R*_s_ (Figs 1c–e and 2). In Northeast China, Northwest China and the Tibetan Plateau, the positive bias of *R*_s_ is dominated by the overestimated *R*_s-clear_ that is highly sensitive to *R*_s_, completely offsetting the overestimated TCC that is slightly sensitive to *R*_s_ (Figs 1c–e and 2).

**Figure 3.**
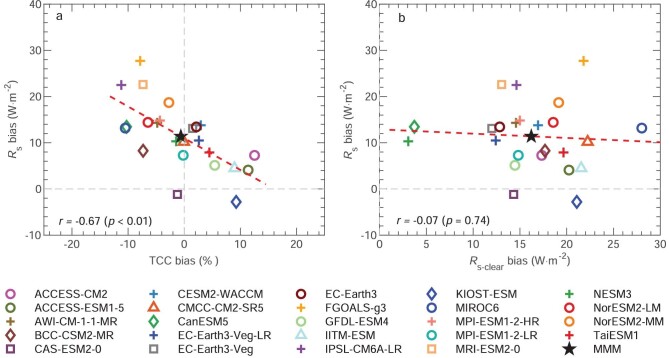
Inter-model relationship of the biases. (a) and (b) Relationship of the simulated biases in surface downward solar radiation (*R*_s_) against those in (a) total cloud cover fraction (TCC) and (b) clear-sky surface downward solar radiation (*R*_s-clear_) for 24 individual model simulations (colored dots) averaged over China from 1961 to 2014. Correlation coefficient (*r*) with a significance level (*p*) between them is shown and their least-square linear fit is plotted as red dash line.

The inter-model relationships of the *R*_s_ bias against the TCC and *R*_s-clear_ biases can also offer additional insights into the possible causes of the *R*_s_ bias (Fig. 3). The *R*_s_ bias clearly demonstrates a strong inter-model correlation with the TCC bias (*r* =* –*0.67, *P* < 0.01), with a larger underestimation of TCC simulation responding to a larger overestimation of *R*_s_ simulation (Fig. 3a), and this relationship is also robust over the grids (Supplementary Fig. S3a). This significant relationship between the *R*_s_ and TCC biases suggests that the inter-model differences may have the same underlying physical drivers as the individual model bias has [22]. Although the inter-model relationship between the *R*_s_ and *R*_s-clear_ biases is weak on the national scale (Fig. 3b), such significantly positive correlations can be seen at most grids in Northwest China and Southeast China (Supplementary Fig. S3b). Most models overestimate *R*_s-clear_ to enhance the positive bias in the simulated *R*_s_ or partly alleviate the effect of the TCC overestimation (Figs 3 and 1c–e).

### Impact of model bias on the *R*_s_ projections

The national mean *R*_s_ simulated in three future SSP scenarios (SSP1-2.6, SSP2-4.5 and SSP5-8.5) show different levels of upward trends with large uncertainties during China's planned 20-year carbon-neutrality period (2050–2069), relative to the last 20 years of historical simulations (1995–2014) (Supplementary Fig. S1a). The systematic biases in TCC and *R*_s-clear_ can cause the historical bias of *R*_s_ revealed above and are bound to greatly affect the future projections of *R*_s_ and their uncertainty. EC based on this physical relationship allow us to constrain the future projections of *R*_s_ and narrow down the projection uncertainty, which can increase our ability and confidence in future solar energy deployment.

**Figure 4.**
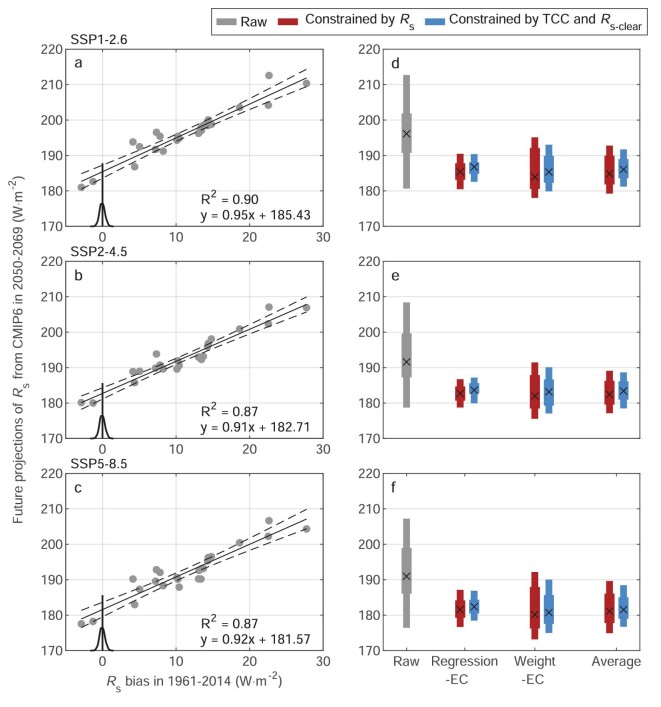
Constraining the model projections of surface downward solar radiation. (a)–(c) Constraining the model projections of surface downward solar radiation (*R*_s_) in three possible future scenarios, i.e. (a) SSP1-2.6, (b) SSP2-4.5 and (c) SSP5-8.5, with the help of the observations. The gray dots show the future *R*_s_ averaged over all grids in China during 2050–2069 versus the historical bias in *R*_s_ during 1961–2014 for the 24 models. Their robust regression fit shown as a gray line represents the constraint relationship for the future *R*_s_, and the dashed lines show the 95% confidence intervals estimated by bootstrap. R^2^ is the goodness-of-fit for the regression. The vertical black line shows that the bias between the simulated and observed *R*_s_ is equal to 0 and its probability density function is inferred from the differences between the bootstrap-resampled averages (see ‘Methods’ section) of the observations during 1961–2014 and their mean. (d)–(f) Comparisons of raw and constrained projections of *R*_s_ in three future scenarios. Four groups of bars are the raw projections of *R*_s_ (gray) averaged over China during 2050–2069, the constrained projections of *R*_s_ using the weight-EC and regression-EC methods (see ‘Methods’ section) and their average of the constraint projections, respectively. The projections are constrained based on the historical bias in *R*_s_ (red) and its combined effect from total cloud cover fraction (TCC) and clear-sky surface downward solar radiation (*R*_s-clear_) (blue), respectively. The mode (×) and confidence intervals (66% and 95%) estimated from the probability density function of the constrained projections are shown over the bar.

Figure 4a–c shows robust linear relationships between the future projections of national mean *R*_s_ during 2050–2069 for three future SSP scenarios and the systematic model bias in *R*_s_ identified above, with a goodness-of-fit of ∼0.90 (*P* < 0.05). Such robust relationships also apply to the grid scale under three future SSP scenarios (Supplementary Fig. S4). Leveraging the robust relationships, Fig. 4d–f describes the constrained future projection of *R*_s_ during 2050–2069 and their uncertainties based on two types of EC methods (see ‘Methods’ section), i.e. one is based on posterior probability weight (weight-EC) and the other is based on regression (regression-EC). Considering the different advantages of the regression-EC and weight-EC methods, we average their constrained projections of *R*_s_ (Fig. 4d–f) for better supporting decision-making in the future. Compared with the raw projections, the constrained projections in 2050–2069 are reduced in the national mean *R*_s_ by ∼5%, i.e. from 196 to 185 W·m^−2^ in SSP1-2.6, 192 to 182 W·m^−2^ in SSP2-4.5 and 191 to 181 W·m^−2^ in SSP5-8.5 (Fig. 4d–f). More importantly, the projection uncertainties of *R*_s_ are reduced by ∼56%, which significantly improves inter-model agreement in *R*_s_ and substantially increases our confidence in the future projections of *R*_s_ (Fig. 4d–f). Moreover, we find that the constraints using the combined effect of the TCC and *R*_s-clear_ biases can account for ∼81% of the projection uncertainties using *R*_s_ (Fig. 4d–f). The constraints are also applied to the grid scale and yield similar results as above.

**Figure 5.**
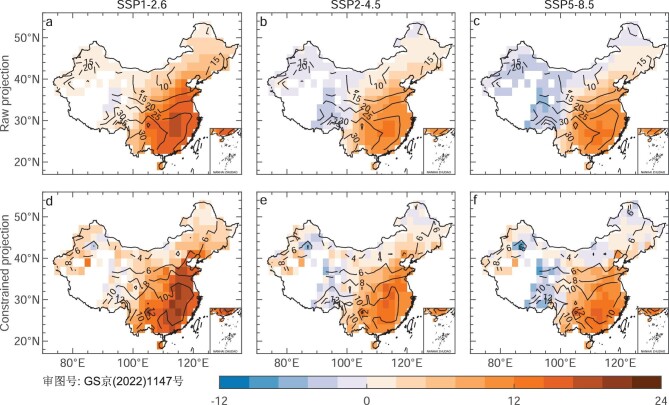
Maps of the constrained future changes in *R*_s_ and their uncertainty. (a)–(f) Future changes (shading; in W·m^−2^) in the 20-year mean of surface downward solar radiation (*R*_s_) during 2050–2069 relative to the 1995–2014 mean from the (a)–(c) raw and (d)–(f) constrained values in three possible future scenarios, i.e. SSP1-2.6, SSP2-4.5 and SSP5-8.5, with the 66% confidence interval shown as a contour. The constraints are done for both periods to derive the future change and the results of the weight-EC and regression-EC are averaged in (d–(f).

Credible projection of *R*_s_ values obtained by the EC with the help of historical observations can yield more realistic estimates for future changes in *R*_s_. Figure 5 shows the spatial patterns of future changes in *R*_s_ calculated as the differences of the constrained simulations between 2050–2069 and 1995–2014. Compared with the raw future change, the constrained result of the CMIP6 MMM *R*_s_ in SSP1-2.6 shows a higher level of brightening during 2050–2069 relative to the 1995–2014 mean in North China and Southeast China with higher power demand (Fig. 5a and d). The constrained change increases by ∼30% in China referenced to the raw projection in SSP1-2.6 (Fig. 5a and d). With increased anthropogenic forcing in SSP2-4.5 and SSP5-8.5, the constrained future changes become weaker brightening in eastern China and more dimming in western China (Fig. 5b, c, e and f). This could imply that low anthropogenic emissions under the carbon-neutrality actions would increase future changes in *R*_s_ and solar energy potential, consequently creating positive feedback for building a climate-resilient society. More importantly, the uncertainties of future changes in *R*_s_ are reduced by 70% in North China and Southeast China and by 30% for the rest of China.

## DISCUSSION

Effectiveness and confidence in the layout of solar PV systems heavily depend on reliable projections of *R*_s_. Considering model bias and scenario uncertainty, the future projections of *R*_s_ in the mid-twenty-first century by the CMIP6 models in three possible future emission scenarios are constrained with the help of historical observations in this study. Results show that future increase in the constrained *R*_s_ is largest in SSP1-2.6, a low-emission scenario, followed by SSP2-4.5 and SSP5-8.5, median- and high-emission scenarios (Fig. 5). According to the linear relationship between *R*_s_ and electrical power by solar (see ‘Methods’ section) [34], the potential electrical power generated by solar in China at SSP1-2.6, SSP2-4.5 and SSP5-8.5 is estimated to be 44.3, 43.6 and 43.3 TW per year on average during 2050–2069, respectively. These results are more favorable for increasing the share of solar energy in the future for phasing out heavily polluting coal as its major energy source, which is conducive to the formation of positive feedback in the producing clean energy—tackling climate change under the carbon-neutrality actions.

In China, there are substantial regional variations in solar power generation potential affected by shortwave radiation, land availability and installation densities, showing a downward trend from northwest to southeast [35,36]. However, most western regions including Xinjiang, Inner Mongolia, Gansu, Qinghai and Tibet with huge solar PV generation potential have relatively low electricity demand and population density [35,37], so it needs huge costs to realize power transmission from west to east. Taking into account the cost of spatial dislocations between the PV power generation potential and electricity demand in China, and the need to improve air quality by reducing coal use in eastern China, the development of PV systems has recently begun to shift from west to east [37]. The higher level of brightening in the future in North China and Southeast China revealed by our constrained projections of *R*_s_ in the low-emission scenario (Fig. 5) directly supports the west-to-east shift of the PV systems, which can make better use of future solar energy resources.

Limitations of our analysis are revealed here for better understanding our results. First, sunshine duration data are recorded by visually reading the burned signals on light-sensitive paper and TCC data are observed based on human eye, so the shift work of different meteorological observers may cause problems on the data homogeneity [38,39]. Our homogenization has removed most large

discontinuities contained in these data, which can minimize the impact of the observational uncertainty. In addition, sparse ground-based observations over complex terrain in the Tibetan Plateau may also lack spatial representation compared to the rest of China [6]. Second, our methods work well to obtain the best estimates and their uncertainty of the national average *R*_s_ projections by building a robust constraint relationship using model and observational data, but are not able to constrain *R*_s_ on those grids without observational data, such as the western Tibetan Plateau (Fig. 5). Third, uncertainties in future projections may suffer from some limitations of different constraint methods. The weight-EC method presents a limitation in the projected *R*_s_ because it weights the models by the posterior probabilities of their historical simulations against the observations and consequently 4 out of 24 models have large weights (Table 1). In contrast, the regression-EC method treats each model as equal and independent, and leverages the robust linear relationship to constrain the projections of *R*_s_. Its result may be disturbed by the models that are significantly inconsistent with the observations or that might share some interdependent modules despite being modified among the models [40], while the weight-EC could overcome these disadvantages [26]. On this basis, our result shown above is averaged from their *R*_s_ projections constrained by the two methods, considering their different advantages and inherent systematic bias of climate models over China, so we believe that the constrained projections are more credible than the raw projections. Finally, while the relationship between *R*_s_ and electrical power by solar is actually complex [41], a simple linear model is able to describe their relationship [34].

In summary, the significant systematic bias in *R*_s_ in the CMIP6 models is identified in this study and its drivers are further revealed to be model biases in simulating TCC and *R*_s-clear_. These biases have significant impacts on future projections of *R*_s_ and their uncertainties from these models. Observation-based EC through this robust relationship largely reduce the projection uncertainties of future *R*_s_ by ∼56% and increase brightening by ∼30% in the future *R*_s_ changes during 2050–2069 in China. The constrained projections of *R*_s_ show a higher level of future brightening in North China and Southeast China, highlighting the need to consider the spatial changes in future *R*_s_ when making policies or decisions associated with future solar energy deployment.

## METHODS

### Observation and model data

Direct measurements of surface downward solar radiation (*R*_s_) are conducted only at ∼100 stations in China. These direct *R*_s_ measurements not only are unevenly located, but also suffer from series inhomogeneity due to instrument aging and instrument sensitivity drift problems [42–44], so that they are

not able to well depict historical *R*_s_ and its change in China. Unlike direct *R*_s_ measurements, sunshine duration (SunDu) was measured at ∼2200 stations from 1961 to 2014. SunDu has been used to derive *R*_s_ (SunDu-derived *R*_s_; Equations (1) and (2)) [6,7] based on the revised Ångström-Prescott equations [45]. The SunDu-derived *R*_s_ avoids series inhomogeneities not only due to instrument aging and instrument sensitivity drift problems contained in direct *R*_s_ measurements, but also due to large-scale instrument replacements in 1990–1993 across China [42,43,45]. The SunDu-derived *R*_s_ can well reproduce monthly *R*_s_ values in China with a bias of 2.19 W·m^−2^ (1.4%) and a standard deviation of 19.32 W·m^−2^ (12.0%) compared with direct *R*_s_ measurements and is able to describe monthly to decadal *R*_s_ variability well [7,42], which has been used as reference data to assess the performance of *R*_s_ in the reanalysis products and climate simulations [27,38,46]. Here, the SunDu-derived *R*_s_ data set at ∼2200 stations in He *et al.* [7] is used as observations for comparisons with the CMIP6 historical all-forcing simulations:

(1) \begin{eqnarray*} {R}_{\rm s} = {\tau }_c \int ( {{\tau }_{c\_dir} + \ {\tau }_{c\_dif}} ) \times {I}_0\ {d}_t, \end{eqnarray*}

(2) \begin{eqnarray*} {\tau }_c = {a}_0\ + {a}_1\left( {\frac{n}{N}} \right) + \ {a}_2{\left( {\frac{n}{N}} \right)}^2, \end{eqnarray*}

where *a*_0_, *a*_1_ and *a*_2_ represent the regression coefficients of the sunshine duration against the *R*_s_ observation; *n* and *N* represent the observed sunshine duration and theoretical sunshine duration, respectively; *τ_c_* is the radiative transmittance due to cloud extinction; *I*_0_ is the solar irradiance at the top of the atmosphere; *t* is the time (in seconds); *τ_c___dir_* and *τ_c___dif_* denote the direct radiation transmittance and the diffuse radiation transmittance under clear skies, respectively, which are calculated through a broad radiative transfer model based on meteorological observations including near-surface air temperature, air pressure and relative humidity and the turbidity coefficient [45].

TCC measured at ∼2200 stations by the China Meteorological Administration (CMA, http://data.cma.cn/en) are used in this study. Noted is that TCC is observed based on the human eye and the number of stations with TCC observations decreased to 800 in 2014. To supplement TCC data for those stations without TCC observations in 2014, we apply inverse distance weighting interpolation to the observations at nearby stations that have a correlation coefficient of >0.7 with the anomaly of the candidate station during 1961–2013 [38,47].

*R*_s_ and TCC data in the CMIP6 experiments (https://esgf-node.llnl.gov/search/cmip6/) are used in this study, including historical all-forcing simulations (HIST; 1961–2014) and future projections in three possible scenarios with different shared socio-economic pathways (SSP1-2.6, SSP2-4.5 and SSP5-8.5; 2015–2099). To ensure an equal weight for different CMIP6 models, the ‘rlilp1f1’ realizations are adopted in this study. There are 30 models providing both historical and future data, 24 out of which are selected by comparing the *R*_s_ anomalies in the HIST simulations with those of observations via the Kolmogorov–Smirnov test at a significance level of 0.05. This method is often used to filter out some models with apparently inappropriate climate simulations [48]. Information of the models used are listed in Table 1.

We describe the clear-sky surface downward solar radiation (*R*_s-clear_) by using the days with TCC of <15% as the clear-sky data [47]. It has been shown that there is little difference in the monthly *R*_s_ climatology under different clear-sky thresholds (e.g. 10% and 15%) with the one under true cloud-free conditions (0%). To reduce the sampling effect of uneven observation sites and ensure spatial consistency between model grids and observation sites, we integrate all the data onto 2° × 2° grid boxes by averaging observations of all the sites within a grid box or bilinearly interpolating model grids. We calculate the national average by weighting the area of each grid.

### Statistical analyses

Mean bias (MB), root-mean-square error (RMSE) and Pearson correlation coefficient (*r*) are used to quantify historical bias in *R*_s_, TCC and *R*_s-clear_ of the CMIP6 historical all-forcing simulations against the observations:

(3) \begin{eqnarray*} {\rm{MB\ }} = \frac{1}{m}{\rm{\ }} \times \mathop \sum \limits_{i {=}1}^m ({S}_i - {O}_i), \end{eqnarray*}

(4) \begin{eqnarray*} {\rm{RMSE\ }} = \sqrt {\frac{1}{m} \times \mathop \sum \limits_{i = 1}^m {{\left( {{S}_i - {O}_i} \right)}}^2}, \end{eqnarray*}

(5) \begin{eqnarray*} r\ = {\rm{\ }}\frac{{\mathop \sum \nolimits_{i {=} 1}^m ( {{S}_i - \bar{S}} )( {{O}_i - \bar{O}})}}{{\sqrt {\mathop \sum \nolimits_{i {=} 1}^m {{( {{S}_i - \bar{S}} )}}^2} \sqrt {\mathop \sum \nolimits_{i {=} 1}^m {{( {{O}_i - \bar{O}} )}}^2}}}, \end{eqnarray*}

where *m* is the number of the *R*_s_, TCC or *R*_s-clear_ data; *S_i_* and *O_i_* denote the *i*^th^ data of the CMIP6 simulations and observations, respectively; $\bar{S}$ and $\bar{O}$ are the mean of the simulations and observations, respectively.

To quantify the sensitivities of *R*_s_ bias to the TCC or *R*_s-clear_ bias, we use partial least squares to statistically exclude the confounding effects of the other variable:

(6) \begin{eqnarray*} {\rho }_{\left( {x,y} \right)|z} = \ \frac{{{r}_{x,y} - \ {r}_{x,z}{r}_{y,z}}}{{\sqrt {\left( {1 - {r}_{x,z}^2} \right)\left( {1 - {r}_{y,z}^2} \right)}}}, \end{eqnarray*}

where ${\rho }_{( {x,y} )|z}$ is the partial correlation coefficient between *x* and *y* after controlling *z; x* represents the *R*_s_ bias; *y* or *z* can be the TCC or *R*_s-clear_ bias; *r* is the Pearson correlation coefficient.

### EC

Two emergent constraints methods are used to constrain future projection: one is based on posterior probability weight (weight-EC) and the other is based on regression (regression-EC).

#### Weight-EC

This method is to estimate a posterior probability through an information–theoretic perspective according to how well the models reproduce the observations [26]. The estimated posterior probability is used for weighting the future projections given the climate models to yield robust constraints assuming a robust linear relationship with historical data. To this end, we first calculate the Kullback–Leibler divergence, also known as relative entropy or information divergence, to measure the asymmetry of the probability distributions of *R*_s_ between the historical simulation of each model and the observation (Equation (7)) [49]. Then, we estimate the weight (*w*) by an information–theoretic distance measure (*l*) between each model and the observation (Equations (8) and (9)), which is also verified by visual comparison between them. The weights for all the models are listed in Table 1. We also identify the robust linear relationship between future projections of *R*_s_ and historical *R*_s_ bias (Fig. 4a–c) and, based on this relationship, we can obtain the probability density functions (PDF) of the constrained *R*_s_ by applying the estimated posterior probability and then use a Gaussian kernel to estimate the mean and uncertainty of the projected *R*_s_:

(7) \begin{eqnarray*} {\Delta }_j = \int O( x )\log \frac{{O(x)}}{{{S}_j(x)}}dx, \end{eqnarray*}

(8) \begin{eqnarray*} {l}_j = {e}^{ - {\Delta }_j}, \end{eqnarray*}

(9) \begin{eqnarray*} {w}_j = \ \frac{{{l}_j}}{{\mathop \sum \nolimits_j {l}_j}}, \end{eqnarray*}

(10) \begin{eqnarray*} \mathop \sum \limits_j {w}_j = \ 1, \end{eqnarray*}

where *O*(*x*) is the PDF of the observation; *s_j_*(*x*) is the PDF of the historical simulation in the *j*^th^ model; ${\Delta }_j$ is the Kullback–Leibler divergence of each model; *l_j_* is the information–theoretic distance describing how well the historical simulation reproduces the observation, which is quantified here as the likelihood of the *j*^th^ model given the observed distribution; *w_j_* is the normalized weight of the *j*^th^ model.

#### Regression-EC

This method is to leverage the robust inter-model relationship (Equation (11)) to constrain the future projection of *R*_s_ with the help of the observation. The parametric uncertainty of the regression model and the observation uncertainty are considered. To account for the parametric uncertainty, we repeat the inter-model regression between future projections of *R*_s_ and model biases of *R*_s_ via 1000-times bootstraps. To estimate the uncertainty of the observation average, we estimate the PDF of the average via 1000-times bootstraps of annual *R*_s_ observations during 1961–2014. The observation uncertainty contained in the *R*_s_ bias can be quantified by the PDF of the observation average:

(11) \begin{eqnarray*} Y\ = \ \alpha X + \ \beta, \end{eqnarray*}

where *Y* is the future projection of *R*_s_ from CMIP6 models in each of three possible future scenarios and *X* is the historical model bias in *R*_s_; $\alpha $ is the regression slope and $\beta $ is the intercept. The value of *Y* as *X* equals 0 is the constrained projection.

To constrain future projections of *R*_s_, we take the bias of 0 and its PDF as input data into each bootstrap regression equation (Equation (11)) to generate a pool of future constrained *R*_s_. As the weight-EC method, we also employ a Gaussian kernel with a bandwidth chosen to minimize the mean integrated squared error of the pool of future constrained *R*_s_, to estimate the mean and uncertainty of the projected *R*_s_. Previous studies [22,50] usually ignore either of them in their EC constraints, thereby weakening the estimated uncertainty.

To verify the validity of the constraint methods, we constrain the recent 20-year mean historical *R*_s_ simulations of the CMIP6 models averaged over the grids with the observations in China during 1995–2014 based on the former period of 1961–1994 and compare the constrained results with the 1995–2014 observations. The comparison shows they match well and the uncertainties are significantly reduced (Supplementary Fig. S5).

### Estimate of the combined effect of TCC and *R*_s-clear_

To estimate the combined effect of TCC and *R*_s-clear_ on *R*_s_ in the observations from 1961 to 2014, a multiple linear regression is applied:

(12) \begin{eqnarray*} {R}_{\rm s} = {\rm{\ }}{b}_0 + \ {b}_1 \cdot {\rm{TCC}} + \ {b}_2 \cdot {R}_{\rm s\hbox{-}{\rm clear}} + \varepsilon, \end{eqnarray*}

where ${b}_1$ and${\rm{\ }}{b}_2$ are the regression coefficients; ${b}_0$ is the constant and $\varepsilon $ is the residual. Then, we repeat the constraints using the combined effect of TCC and *R*_s-clear_, instead of *R*_s_, based on the weight-EC and regression-EC methods.

### Estimate of electrical power generated by solar

A model [34] to estimate electrical power (EP) generated by solar is used to show its linear relationship with *R*_s_, as the following:

(13) \begin{eqnarray*} {\rm{EP\ }} = \ S\ \times \ [ {\eta \times ( {1 - {\alpha }_p} )} ] \times \ {A}_\rho, \end{eqnarray*}

where *S* represents the annual average *R*_s_ in China during 2050–2069; $\eta $ is the average conversion efficiency set at 26.9% in 2060 where air temperature plays a role [37]; ${\alpha }_p$ is the panel albedo assumed to be 0.1 [34]; *A_p_* represents the suitable land area for PV power generation, which is set at 0.99 million km^2^ as suggested by Qiu *et al.* [35] based on the 2015 situation in China.

## DATA AVAILABILITY

Climate model outputs from CMIP6 are publicly available at https://esgfnode.llnl.gov/search/cmip6/. Routine meteorological observations at ∼2200 stations are obtained from the CMA (http://data.cma.cn/en). The homogenized meteorological observations and ground-based *R*_s_ from sunshine duration used in this study are available upon reasonable request to the corresponding author. The code is available upon request to the corresponding author.

## Supplementary Material

nwac242_Supplemental_File

## References

[1] Gernaat DEHJ , de Boer HS, Daioglou V et al. Climate change impacts on renewable energy supply. Nat Clim Chang 2021; 11: 119–25. 10.1038/s41558-020-00949-9

[2] Tong D , Farnham DJ, Duan L et al. Geophysical constraints on the reliability of solar and wind power worldwide. Nat Commun 2021; 12: 6146. 10.1038/s41467-021-26355-z 34686663 PMC8536784

[3] Wang YL , Duan CY, Lv P et al. Printing strategies for scaling-up perovskite solar cells. Natl Sci Rev 2021; 8: 64–87. 10.1093/nsr/nwab075 PMC8363337 34691715

[4] Global Energy Interconnection Development and Cooperation Organization . *China's Carbon Neutrality Research Report by 2060* (in Chinese). Beijing: China Electric Power Press, 2021.

[5] Wild M , Gilgen H, Roesch A et al. From dimming to brightening: decadal changes in solar radiation at Earth's surface. Science 2005; 308: 847–50. 10.1126/science.1103215 15879214

[6] He Y , Wang K. Variability in direct and diffuse solar radiation across China from 1958 to 2017. Geophys Res Lett 2020; 47: e2019GL084570. 10.1029/2019GL084570

[7] He Y , Wang KC, Zhou CL et al. A revisit of global dimming and brightening based on the sunshine duration. Geophys Res Lett 2018; 45: 4281–9. 10.1029/2018GL077424

[8] Wild M . Enlightening global dimming and brightening. Bull Amer Meteor Soc 2012; 93: 27–37. 10.1175/BAMS-D-11-00074.1

[9] Wang Y , Wild M. A new look at solar dimming and brightening in China. Geophys Res Lett 2016; 43: 11777–85. 10.1002/2016GL071009

[10] Wang ZL , Wang CS, Yang S et al. Evaluation of surface solar radiation trends over China since the 1960 s in the CMIP6 models and potential impact of aerosol emissions. Atmos Res 2022; 268: 105991. 10.1016/j.atmosres.2021.105991

[11] Wild M . The global energy balance as represented in CMIP6 climate models. Clim Dyn 2020; 55: 553–77. 10.1007/s00382-020-05282-7 32704207 PMC7366598

[12] Riahi K , Grubler A, Nakicenovic N. Scenarios of long-term socio-economic and environmental development under climate stabilization. Technol Forecast Soc Chang 2007; 74: 887–935. 10.1016/j.techfore.2006.05.026

[13] Gutschow J , Jeffery ML, Gunther A et al. Country-resolved combined emission and socio-economic pathways based on the representative concentration pathway (RCP) and shared socio-economic pathway (SSP) scenarios. Earth Syst Sci Data 2021; 13: 1005–40. 10.5194/essd-13-1005-2021

[14] Huang P , Ying J. A multimodel ensemble pattern regression method to correct the tropical pacific SST change patterns under global warming. J Clim 2015; 28: 4706–23. 10.1175/JCLI-D-14-00833.1

[15] Bracegirdle TJ , Stephenson DB. On the robustness of emergent constraints used in multimodel climate change projections of arctic warming. J Clim 2012; 26: 669–78. 10.1175/JCLI-D-12-00537.1

[16] Whetton P , Macadam I, Bathols J et al. Assessment of the use of current climate patterns to evaluate regional enhanced greenhouse response patterns of climate models. Geophys Res Lett 2007; 34: L14701. 10.1029/2007GL030025

[17] Dai A , Bloecker CE. Impacts of internal variability on temperature and precipitation trends in large ensemble simulations by two climate models. Clim Dyn 2019; 52: 289–306. 10.1007/s00382-018-4132-4

[18] Hawkins E , Sutton R. The potential to narrow uncertainty in regional climate predictions. Bull Amer Meteor Soc 2009; 90: 1095–108. 10.1175/2009BAMS2607.1

[19] Hall A , Cox P, Huntingford C et al. Progressing emergent constraints on future climate change. Nat Clim Chang 2019; 9: 269–78. 10.1038/s41558-019-0436-6

[20] Brient F . Reducing uncertainties in climate projections with emergent constraints: concepts, examples and prospects. Adv Atmos Sci 2020; 37: 1–15. 10.1007/s00376-019-9140-8

[21] Cox PM , Huntingford C, Williamson MS. Emergent constraint on equilibrium climate sensitivity from global temperature variability. Nature 2018; 553: 319–22. 10.1038/nature25450 29345639

[22] Lin YL , Dong WH, Zhang MH et al. Causes of model dry and warm bias over central U.S. and impact on climate projections Nat Commun 2017; 8: 881. 10.1038/s41467-017-01040-2 29026073 PMC5638845

[23] Shiogama H , Watanabe M, Kim H et al. Emergent constraints on future precipitation changes. Nature 2022; 602: 612–6. 10.1038/s41586-021-04310-8 35197617

[24] Thackeray CW , Hall A, Norris J et al. Constraining the increased frequency of global precipitation extremes under warming. Nat Clim Chang 2022; 12: 441–8. 10.1038/s41558-022-01329-1

[25] Liang YX , Gillett NP, Monahan AH. Emergent constraints on CMIP6 climate warming projections: contrasting Cloud- and Surface temperature-based constraints. J Clim 2022; 35: 1809–24. 10.1175/JCLI-D-21-0468.1

[26] Brient F , Schneider T. Constraints on climate sensitivity from space-based measurements of low-cloud reflection. J Clim 2016; 29: 5821–35. 10.1175/JCLI-D-15-0897.1

[27] Freychet N , Tett SFB, Bollasina M et al. The local aerosol emission effect on surface shortwave radiation and temperatures. J Adv Model Earth Syst 2019; 11: 806–17. 10.1029/2018MS001530

[28] Li J , You Q, He B. Distinctive spring shortwave cloud radiative effect and its inter-annual variation over southeastern China. Atmos Sci Lett 2020; 21: e970. 10.1002/asl.970

[29] Cherian R , Quaas J. Trends in AOD, clouds, and cloud radiative effects in satellite data and CMIP5 and CMIP6 model simulations over aerosol source regions. Geophys Res Lett 2020; 47: e2020GL087132. 10.1029/2020GL087132

[30] Li ZQ , Guo JP, Ding AJ et al. Aerosol and boundary-layer interactions and impact on air quality. Natl Sci Rev 2017; 4: 810–33. 10.1093/nsr/nwx117

[31] Boers R , Brandsma T, Siebesma AP. Impact of aerosols and clouds on decadal trends in all-sky solar radiation over the netherlands (1966–2015). Atmos Chem Phys 2017; 17: 8081–100. 10.5194/acp-17-8081-2017

[32] Moseid KO , Schulz M, Storelvmo T et al. Bias in CMIP6 models compared to observed regional dimming and brightening trends (1961–2014). Atmos Chem Phys 2020; 20: 16023–40. 10.5194/acp-20-16023-2020

[33] Wang Z , Lin L, Xu Y et al. Incorrect asian aerosols affecting the attribution and projection of regional climate change in CMIP6 models. npj Clim Atmos Sci 2021; 4: 2443. 10.1038/s41612-020-00159-2

[34] Li Y , Kalnay E, Motesharrei S et al. Climate model shows large-scale wind and solar farms in the sahara increase rain and vegetation. Science 2018; 361: 1019–22. 10.1126/science.aar5629 30190404

[35] Qiu T , Wang L, Lu Y et al. Potential assessment of photovoltaic power generation in China. Renew Sust Energy Rev 2022; 154: 111900. 10.1016/j.rser.2021.111900

[36] Yan J , Yang Y, Elia Campana P et al. City-level analysis of subsidy-free solar photovoltaic electricity price, profits and grid parity in China. Nat Energy 2019; 4: 709–17. 10.1038/s41560-019-0441-z

[37] Lu X , Chen S, Nielsen CP et al. Combined solar power and storage as cost-competitive and grid-compatible supply for China's future carbon-neutral electricity system. Proc Natl Acad Sci USA 2021; 118: e2103471118. 10.1073/pnas.2103471118 34635590 PMC8594571

[38] He Y , Wang K, Feng F. Improvement of ERA5 over ERA-Interim in simulating surface incident solar radiation throughout China. J Clim 2021; 34: 3853–67. 10.1175/JCLI-D-20-0300.1

[39] Xu J , Masuda K, Ishigooka Y et al. Estimation and verification of daily surface shortwave flux over China. J Meteorol Soc Japan 2011; 89A: 225–38. 10.2151/jmsj.2011-A14

[40] Jebeile J , Crucifix M. Multi-model ensembles in climate science: mathematical structures and expert judgements. Studies in History and Philosophy of Science Part A 2020; 83A: 44–52. 10.1016/j.shpsa.2020.03.001 32958280

[41] Müller B , Wild M, Driesse A et al. Rethinking solar resource assessments in the context of global dimming and brightening. Sol Energy 2014; 99: 272–82. 10.1016/j.solener.2013.11.013

[42] Wang K , Ma Q, Li Z et al. Decadal variability of surface incident solar radiation over China: observations, satellite retrievals, and reanalyses. J Geophys Res Atmos 2015; 120: 6500–14. 10.1002/2015JD023420

[43] Wang K . Measurement biases explain discrepancies between the observed and simulated decadal variability of surface incident solar radiation. Sci Rep 2015; 4: 6144. 10.1038/srep06144 PMC4139940 25142756

[44] Tang WJ , Yang K, Qin J et al. Solar radiation trend across China in recent decades: a revisit with quality-controlled data. Atmos Chem Phys 2011; 11: 393–406. 10.5194/acp-11-393-2011

[45] Yang K , Koike T, Ye BJA et al. Improving estimation of hourly, daily, and monthly solar radiation by importing global data sets. Agric For Meteorol 2006; 137: 43–55. 10.1016/j.agrformet.2006.02.001

[46] Zhou C , He Y, Wang K. On the suitability of current atmospheric reanalyses for regional warming studies over China. Atmos Chem Phys 2018; 18: 8113–36. 10.5194/acp-18-8113-2018

[47] Yang S , Wang XL, Wild M. Causes of dimming and brightening in China inferred from homogenized daily clear-sky and all-sky in situ surface solar radiation records (1958–2016). J Clim 2019; 32: 5901–13. 10.1175/JCLI-D-18-0666.1

[48] Zhou CL , Chen DL, Wang KC et al. Conditional attribution of the 2018 summer extreme heat over northeast China: roles of urbanization, global warming, and warming-induced circulation changes. Bull Am Meteorol Soc 2020; 101: S71–6. 10.1175/BAMS-D-19-0197.1

[49] Burnham KP , Anderson DR. Model Selection and Multimodel inference: A practical Information-Theoretic Approach. New York: Springer, 2002.

[50] Chen XL , Zhou TJ, Wu PL et al. Emergent constraints on future projections of the western north Pacific subtropical high. Nat Commun 2020; 11: 2802. 10.1038/s41467-020-16631-9 32499522 PMC7272422

