# Supplementary material for: Constrained future brightening of solar radiation and its implication for China's solar power
Source: Natl Sci Rev. 2022 Oct 29;10(1):nwac242. doi: 10.1093/nsr/nwac242 (PMC9840459; doi:10.1093/nsr/nwac242)
Supplement: nwac242_Supplemental_File [file nwac242_supplemental_file.docx]

Supplementary Materials for

**Constrained future brightening of surface downward solar radiation and its implication for China's solar power**

Yanyi He^1^, Kun Yang^1,2*^, Martin Wild^3^, Kaicun Wang^4^, Dan Tong^1^, Changkun Shao^1^, and Tianjun Zhou^5^

*Corresponding author. Email: [yangk@tsinghua.edu.cn](mailto:yangk@tsinghua.edu.cn)

**This PDF file includes:**

Figs. S1 to S5


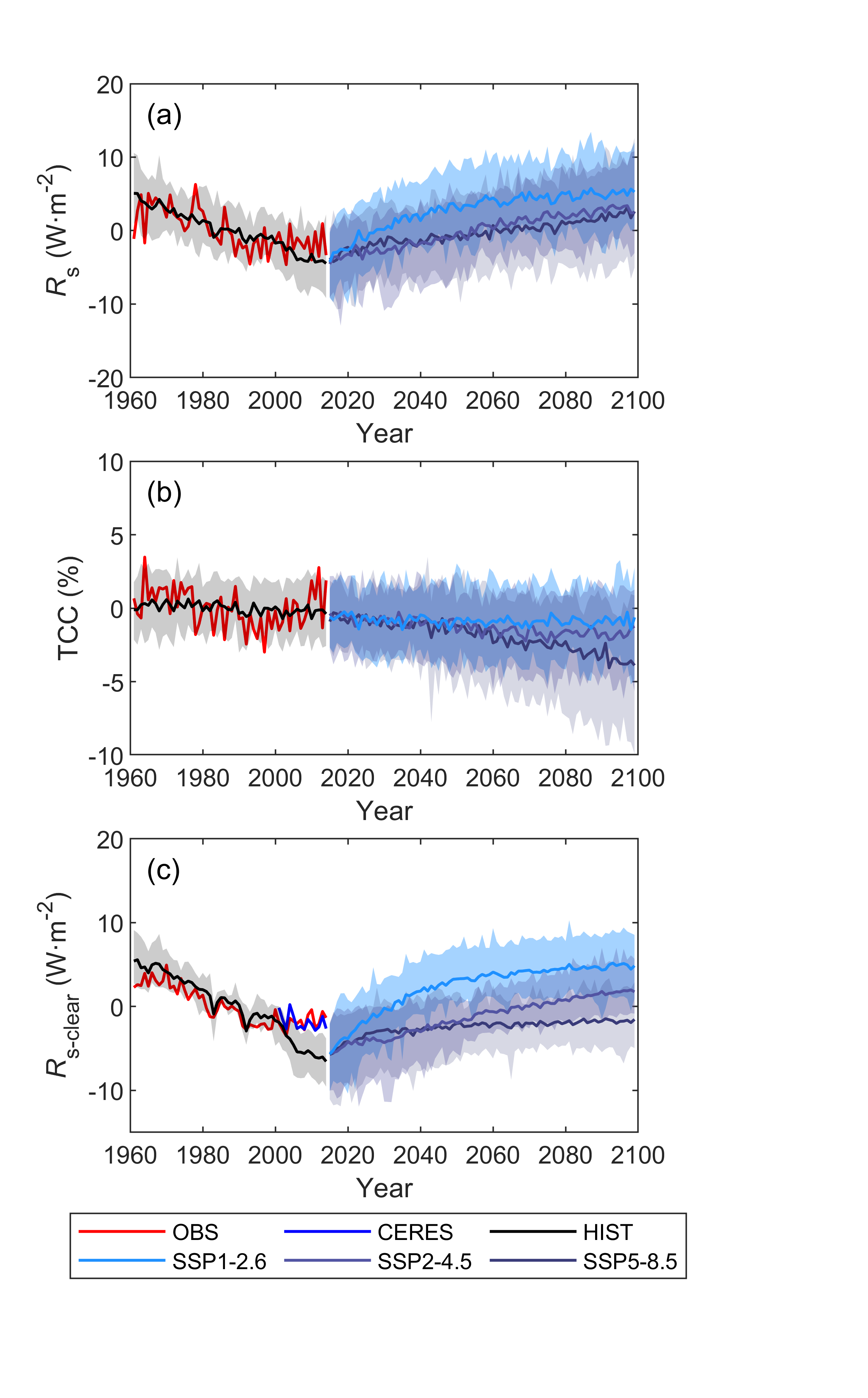


**Figure S1. Time series of observations and climate simulations**. **a-c**, Time series of the (**a**) surface downward solar radiation (*R*_s_), (**b**) total cloud cover fraction (TCC) and (**c**) clear-sky surface downward solar radiation (*R*_s-clear_) anomalies averaged over China from ground-based observation (OBS, red line) during 1961-2014, the CMIP6 historical all-forcing simulations (HIST, black line) during 1961-2014, and the CMIP6 future simulations in three possible future scenarios, i.e., SSP1-2.6, SSP2-4.5 and SSP5-8.5 during 2015-2099. Shading indicates the 5%-95% model spread. The *R*_s-clear_ anomaly from the CERES satellite product during 2001-2014 are shown in blue line. All the anomalies except CERES *R*_s_ are calculated referenced to the 1961-2014 mean. CERES *R*_s_ anomalies are referenced to the 2001-2014 mean and then shifted to the mean of OBS *R*_s_ anomalies during 2001-2014 for visual comparison. Different reference periods are applied and yielded similar results.


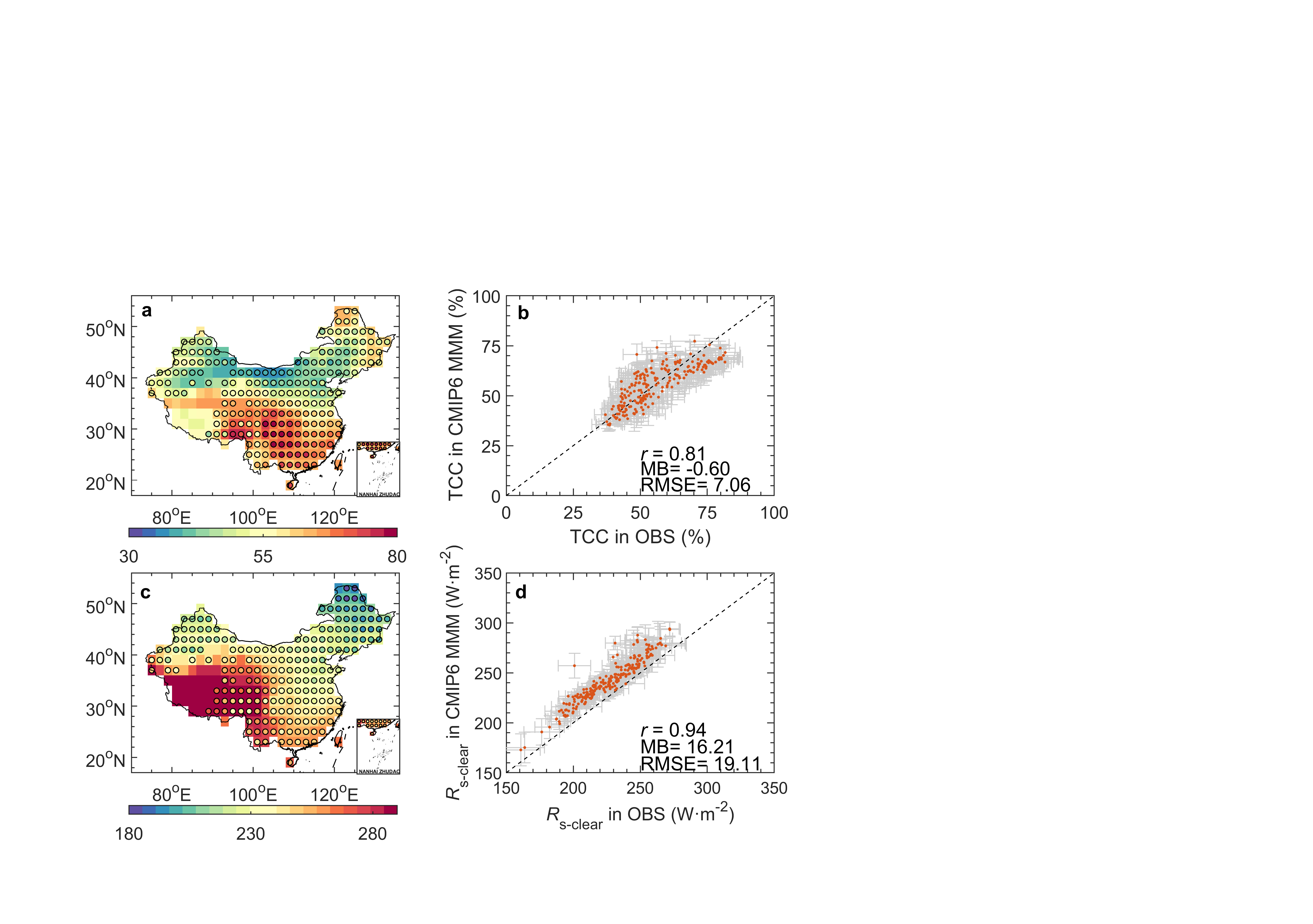


**Figure S2**. **The climatology in TCC and *R*_s-clear_ of observations and climate simulations. a** and **c**, Same as Figure 1a, but for (**a**) total cloud cover fraction (TCC, in %) and (**b**) clear-sky surface downward solar radiation (*R*_s-clear_, in W·m^-2^), respectively. **b** and **d**, Same as Figure 1b, but for (**b**) TCC and (**d**) *R*_s-clear_, respectively.


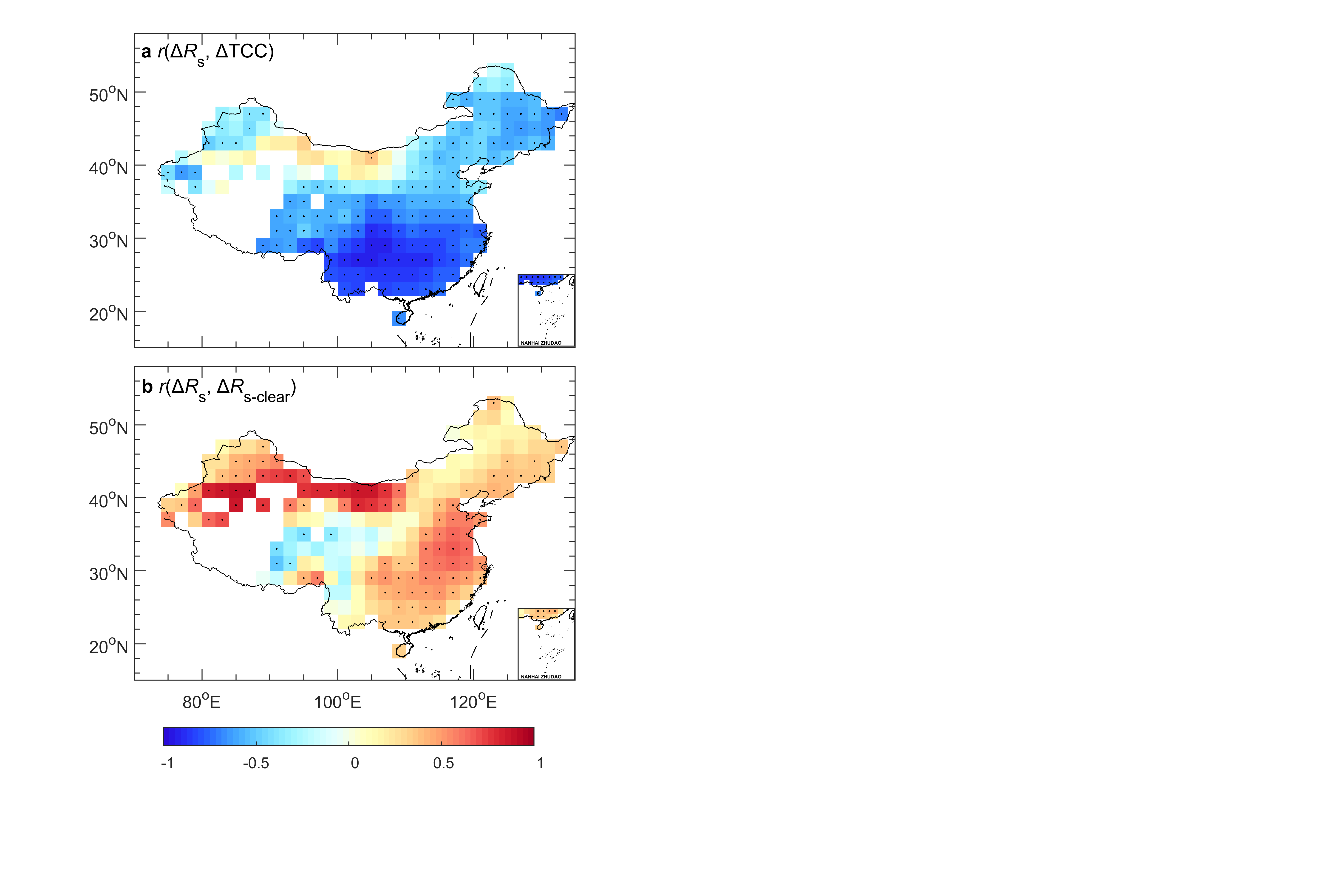


**Figure S3. Correlation of the simulated biases in *R*_s_ for 24 individual model simulations. a-b,** Map of correlation coefficients (shading) of the simulated biases between surface downward solar radiation (∆*R*_s_) and **(a)** total cloud cover (∆TCC) and **(b)** clear-sky surface downward solar radiation (∆*R*_s-clear_) for 24 individual model simulations. Black dots indicate a significance level of 0.05.


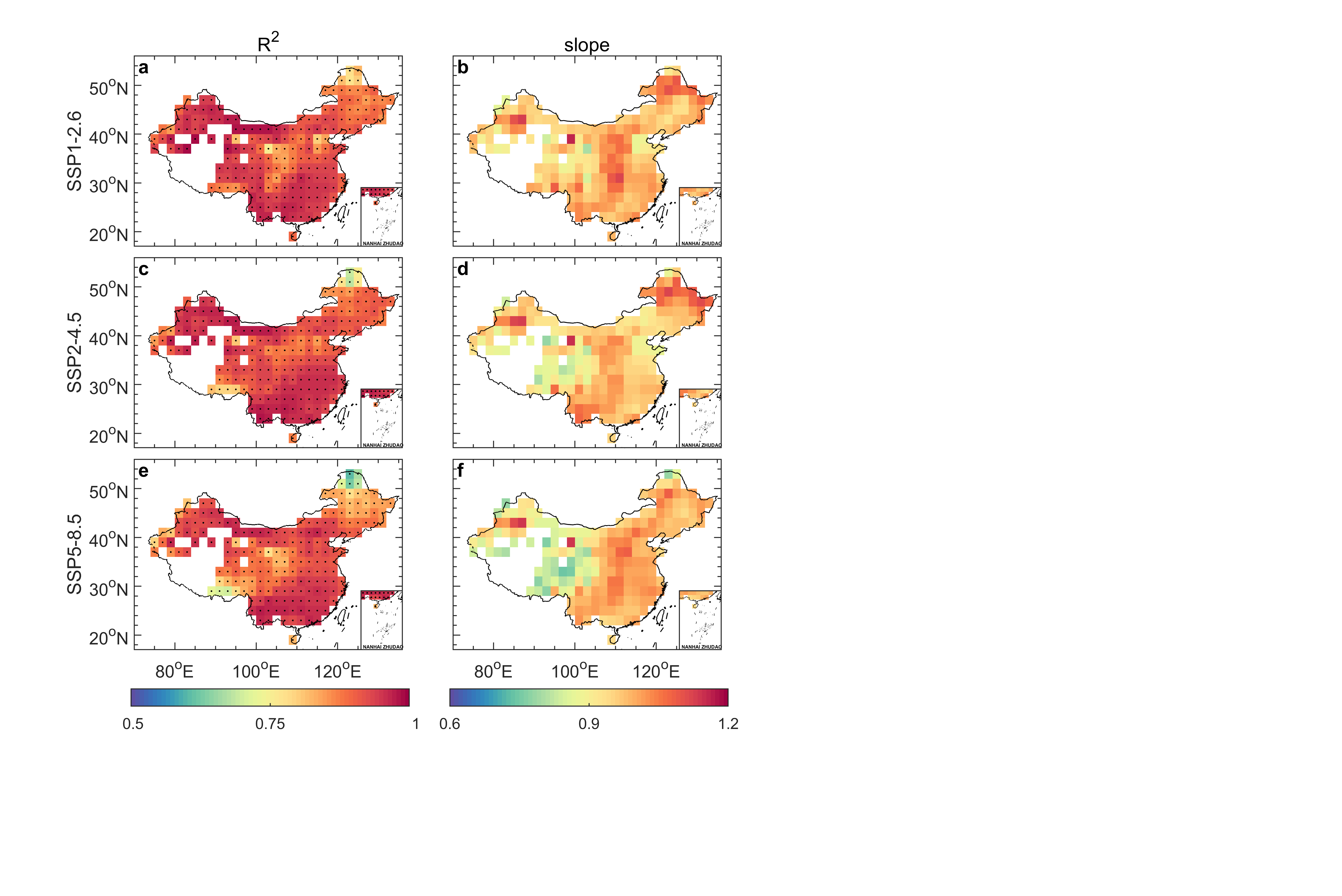


**Figure S4. Maps of the goodness-of-fit (R^2^) and slope for the constrained relationship in three possible future scenarios.** The constrained relationship based on the national average is shown in Figure 4a-c in the main text. These maps show that the relationships over grids are consistent with those based on the national average. Black dots in left panels indicate a significance level of 0.05.


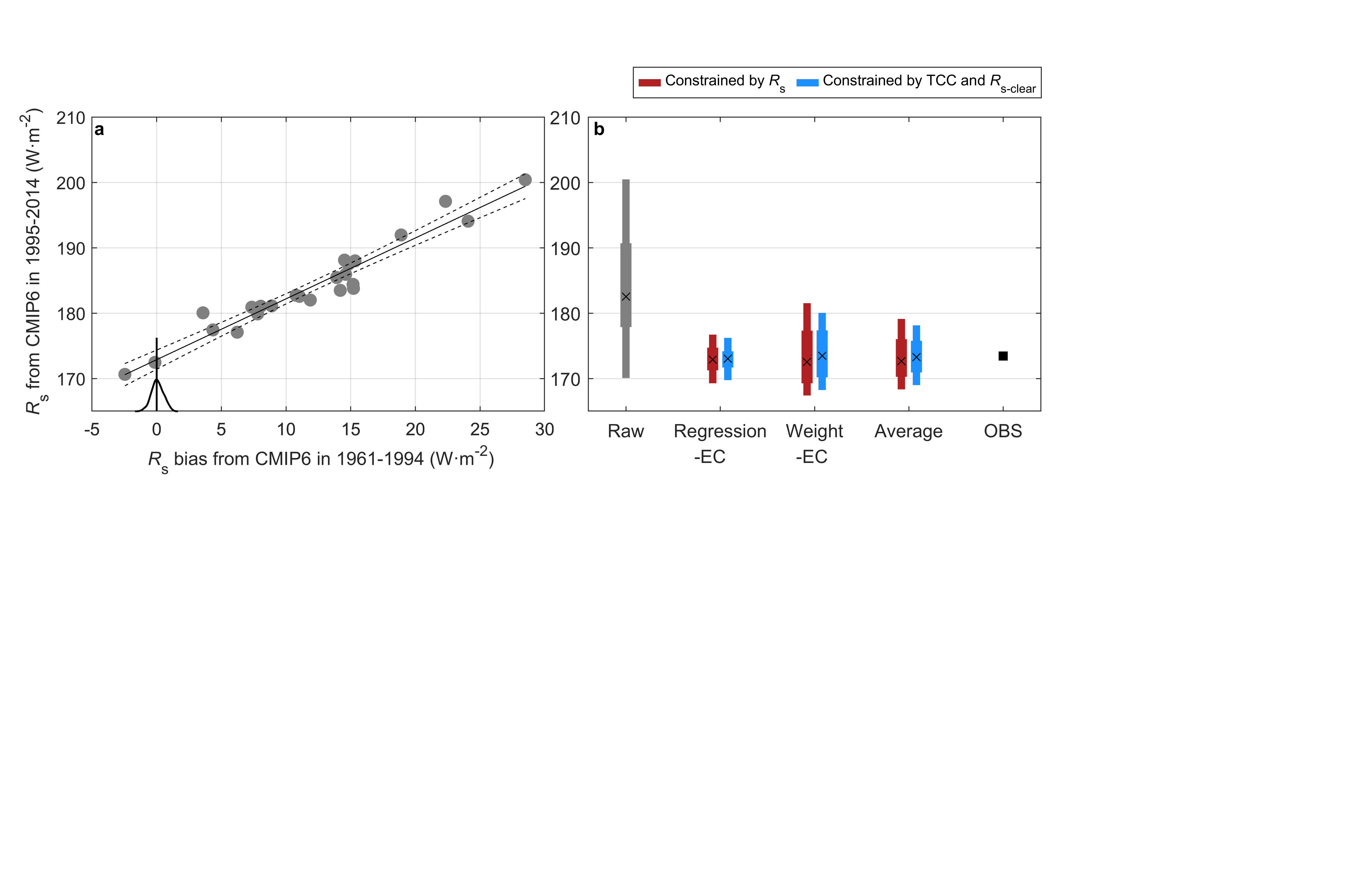


**Figure S5. Validation of the constraint methods. a**, Same as Figure 4a, but constraining the recent 20-year mean historical simulations of *R*_s_ averaged over the grids with the observations in China during 1995-2014 based on the former period of 1961-1994, for the validation using the 1995-2014 observations. **b**, Comparisons of raw and constrained simulations of *R*_s_ with the observations during 1995-2014. Five groups of bars are the raw simulations of *R*_s_ (grey), the constrained simulations of *R*_s_ using the weight-EC and regression-EC constraint methods (see the “Methods” section), their average of the constraint simulations, and *R*_s_ observation averaged over China during 1995-2014 (black square), respectively. The simulations are constrained based on the historical bias in *R*_s_ (red), and its combined effect from total cloud cover fraction (TCC) and clear-sky surface downward solar radiation (*R*_s-clear_) (blue), respectively. The mode (×) and confidence intervals (66% and 95%) estimated from the probability density function of the constrained simulations are shown over the bar.
